# Correlations of intestinal microorganisms with liver and immune functions of patients with human immunodeficiency virus and hepatitis B virus coinfection

**DOI:** 10.4314/ahs.v23i3.53

**Published:** 2023-09

**Authors:** Yong Deng, Ke Yang, Guoqiang Zhou, Ning Wang, Chun Liu, Zhong Chen

**Affiliations:** Department of Acquired Immunodeficiency Syndrome, The First Hospital of Changsha, Changsha 410005, Hunan Province, China

**Keywords:** Intestinal flora, liver function, inflammatory factor, immune, human immunodeficiency virus, hepatitis B virus

## Abstract

**Objective:**

Human immunodeficiency virus (HIV) and hepatitis B virus (HBV) coinfection has threatened the survival of HIV-infected people. To explore the correlations of intestinal microorganisms with liver and immune functions of patients with HIV/HBV coinfection.

**Methods:**

Eighty-six patients positive for HIV antibody and HBV surface antigen diagnosed from January 2018 to June 2020 were selected as HIV/HBV coinfection group. Another 86 patients positive for HBV surface antigen and 86 healthy people were selected as HBV infection and control groups, respectively. The correlations of intestinal flora with liver function, inflammatory indices and immune cells were explored through Pearson's analysis.

**Results:**

Compared with control group, the proportions and numbers of T lymphocytes (CD3^+^), helper T lymphocytes (CD4^+^), cytotoxic T lymphocytes (CD8^+^), CD4^+^/CD8^+^ and natural killer (NK) cells decreased in HIV/HBV coinfection group (P<0.05). IL-2, IL-6, IL-17, ALT, AST, GGT, DBiL and TDBi levels were correlated negatively with *Bifidobacterium, Lactobacillus* and *Bacteroides* numbers, but positively with *Enterobacter* and *Enterococcus* numbers (P<0.05). IL-10 level and proportions of CD3^+^, CD4^+^, CD8^+^, CD4^+^/CD8^+^ and NK cells were correlated positively with *Bifidobacterium, Lactobacillus* and *Bacteroides* numbers, but negatively with *Enterobacter* and *Enterococcus* numbers (P<0.05).

**Conclusion:**

HIV aggravates the liver damage and immuno-inflammatory response in HBV patients.

## Introduction

Human immunodeficiency virus (HIV) infection is a major public health issue worldwide. There have been about 36.7 million people infected with HIV around the world as of the end of 2019, with 2.1 million new cases in the same year^1^. In China, about 958 thousand people survived with HIV/acquired immunodeficiency syndrome (AIDS) at the end of October 20 1 9^2^. Hepatitis B virus (HBV), which is mainly transmitted through blood transfusion, acupuncture and drug abuse, can lead to viral hepatitis B^3^. It has the same route of infection as HIV, so HIV/HBV coinfection occurs easily^4^. WHO reports that besides Mycobacterium tuberculosis, HIV/HBV coinfection is most common in HIV-infected people, which has become the main threat to the survival of HIV-infected patients^5^. Approximately 3 million HIV-infected people worldwide are complicated with HBV infection, accounting for 5-20%. The mortality rate of patients with HIV/HBV coinfection is higher than that of patients with HIV or HBV infection alone^6^. Despite the great improvement in the prognosis of AIDS patients after antiviral therapy, there is a complex interaction between HIV and HBV, leading to diverse courses of disease in co-infected people and increasing the difficulty of treatment^7^. HIV results in the depletion and functional decline of immune cells, leading to persistent high-level viremia upon hepatitis C and aggravating the damage of HBV to the liver. As a result, acute infection develops into chronic infection easily, so hepatic fibrosis progresses more rapidly in patients with HIV/HBV coinfection than that in patients with HBV infection alone^8^.

The intestinal micro-ecosystem is the largest reservoir of bacteria in human body, which plays an important regulatory role in metabolism and immunity^9^. The intestinal flora has a close correlation with a variety of biological mechanisms of the liver. Currently, intestinal microecology has been highlighted in the research on liver diseases^10^. After entering the body, HIV massively reproduces mainly in intestinal lymphatic tissues, attacks CD4^+^ T cells, and releases various pro-inflammatory factors. As a result, the intestinal mucosal barrier is weakened, and the intestinal symbiotic bacteria and their products induce the host to produce series of effector molecules^11^. It has previously been reported that the intestinal flora disturbance has diverse characteristics in different stages of HBV infection^12^. With the increase in severity, the intestinal flora exhibits progressive and structural disorders, and the overall community abundance significantly reduces.

In the present study, therefore, the structure of intestinal flora in patients with HIV/HBV coinfection was analysed, and the correlations of intestinal microorganisms with liver function and immune function were explored, aiming to provide novel insights into the exploration of new therapies.

## Materials and methods

### General data

A total of 86 patients positive for HIV antibody and HBV surface antigen diagnosed in our hospital from January 2018 to June 2020 were selected as HIV/HBV coinfection group. Another 86 patients positive for HBV surface antigen but negative for HIV antibody treated in our hospital in the same period were selected through convenience sampling as HBV infection group. Besides, another 86 healthy people receiving physical examination in our hospital in the same period were selected as control group.

All patients enrolled met the following inclusion criteria: (1) those aged 20-60 years old, (2) those with normal blood routine and occult blood test results, (3) those without infectious diseases such as tuberculosis, hepatitis C and typhoid fever, (4) those without hepatic failure, liver cirrhosis and liver cancer, (5) those without taking antibiotics, micro-ecological modulators, sedatives and hypnotics in the past 4 weeks, and (6) those without other diseases affecting the immune system. This study was approved by the medical ethics committee of our hospital (No. 20171215-3256), and all patients signed the informed consent.

### Detection of HBV surface antigen

Fasting venous blood (10 mL) was collected into anticoagulant-free dry tubes in the early morning, and centrifuged at 3,500 rpm for 5 min to separate the serum. Then 2-3 mL of serum was taken to detect HBV surface antigen by enzyme-linked immunosorbent assay (ELISA) in strict accordance with the instructions of kit (Abcam, USA). Colloidal gold immunochromatographic assay was performed for those positive for HBV surface antigen according to the kit's instructions (Thermo Fisher Scientific, USA).

### Detection of serum biochemical indices

Serum (3 mL) was taken to detect alanine aminotransferase (ALT), aspartate aminotransferase (AST), gamma-glutamyl transpeptidase (GGT), direct bilirubin (DBiL) and total direct bilirubin (TDBi) using Olympus AU2700 automatic biochemical detector (Japan), and to measure interleukin-1 (IL-2), IL-6, IL-10 and tumor necrosis factor-α (TNF-α) by ELISA. The kits were purchased from Shanghai BioRui Co., Ltd. (China), and usein strict accordance with the instructions.

### Detection of lymphocyte subsets

The remaining serum was used to detect T lymphocyte subsets and natural killer (NK) cells by flow cytometry. Specifically, 20 [xL each of three fluorescent antibodies CD3-PC5, CD4-FiTC and CD8-PE (Thermo Fisher Scientific, USA) were added into T lymphocyte subset detection tubes, while CD3-FiTC/CD (16+56)-PE dual-colour fluorescent antibody (Thermo Fisher Scientific, USA) was added into the NK cell detection tubes. Then 100 µL of anticoagulant blood was added, mixed evenly and labeled at room temperature for 15 min. Later, 600 µL of hemolytic agent A (Thermo Fisher Scientific, USA) was added and shaken for 7 s, and then 260 µL of hemolytic agent B (Thermo Fisher Scientific, USA) was added and mixed evenly. After the red blood cells were lysed, the blood was centrifuged at 1,000 rpm for 5 min, the supernatant was discarded, and 4 mL of PBS was added and mixed evenly again, followed by centrifugation at 1,000 rpm for 5 min. After the supernatant was discarded, 500 µL of PBS was added and mixed evenly, and FACSCalibur flow cytometer (BD, USA) was conducted to detect the percentages of T lymphocytes (CD3^+^), helper T lymphocytes (CD4^+^), cytotoxic T lymphocytes (CD8^+^) and NK cells. The absolute value of each lymphocyte subset was calculated as follows: Absolute value of lymphocyte subset (cells/µL) = percentage of lymphocyte subset × absolute value of lymphocytes (detected using a globulimeter, cells/µL). CD3^+^CD4^-^CD8^-^ T lymphocyte count (percentage/absolute value) was calculated as follows: CD3^+^ T lymphocyte count - CD4^+^ T lymphocyte count - CD8^+^ T lymphocyte count.

### Detection of intestinal flora

About 1 g of fresh feces naturally excreted were harvested in each group, and weighed on an electronic balance, from which total DNA was extracted using fecal DNA extraction kit (Qiagen, Germany). The primers for genomic DNA of Bifidobacterium, Lactobacillus, Bacteroides, Enterobacter and Enterococcus were designed using Primer Premier 5.0 software, and amplified using real-time PCR kits (Thermo Fisher Scientific, USA). The cycle threshold n was obtained, and log N/g was calculated. Replicate wells were set for each sample. The primer sequences are listed in Table 1.

**Table 1:** Primer sequences and product lengths

Bacterium	Primer sequence	Product length (bp)
*Bifidobacterium*	Upstream 5′-GTTCGCGTAAACACTGAATACTGA-3′ Downstream 5′-TCGCGTCCCCGAACCTTATACTGTT-3	342
*Lactobacillus*	Upstream 5′-CAGGGGGAACTGTGCGCGATGATT-3′ Downstream 5′-CAAGCCCAATCACTTCCGCGATTGT-3′	156
*Bacteroides*	Upstream 5′-CTCCCGCGGCAATCGCTGTAAAGCG-3′ Downstream 5′-CACCCGCGGCAAACTTCCACATTAA-3′	200
*Enterobacter*	Upstream 5′-TCGCTCGCGCTCTGAACGTCCACTG-3′ Downstream 5′-CGTCCGACGAAGCCGCGTCACTCCAC-3′	243
*Enterococcus*	Upstream 5′-GTAACTGCGCGACTTAACGAATCGG-3′ Downstream 5′-GCTAAGAACATCCCGCGTGCGAGGAG-3′	341

### Statistical analysis

SPSS20.0 software was used for statistical analysis (IBM Inc., USA). Numerical data were expressed as rate (%), and the x^2^ test was performed for intergroup comparison. Pearson's correlation analysis was conducted. P<0.05 was considered to be statistically significant.

## Results

### Baseline clinical data

The HIV/HBV coinfection group included 54 males and 32 females aged 24-69 years old, with an average of (35.63±8.34) years old. The HBV infection group consisted of 56 males and 30 females aged 20-67 years old, with an average of (36.32±8.16) year old. The control group included 60 males and 26 females aged 22-65 years old, with an average of (36.83±7.92) years old. There were no significant differences in gender and age among the three groups (P>0.05).

### Liver function

Compared with those in control group, the levels of liver function indices (ALT, AST, GGT, DBiL and TDBi) significantly rose in HBV infection group (P<0.05). The levels of these indices were significantly higher in HIV/HBV coinfection group than those in control group and HBV infection group (P<0.05) (Table 2).

**Table 2:** Liver function indices (x̅±s)

Group	ALT (U/L)	AST (U/L)	GGT (U/L)	DBiL (µmol/L)	TDBi (µmol/L)
Control (n=86)	36.38±2.39	32.95±3.24	42.54±5.34	10.34±2.41	6.39±3.56
HBV infection (n=86)	64.39±5.34^a^	84.34±6.49^a^	68.39±5.59^a^	22.49±3.59^a^	58.38±12.56^a^
HIV/HBV coinfection (n=86)	78.39±8.38^a^^,^^b^	123.38±12.34^a^^,^^b^	93.39±6.49^a^^,^^b^	34.94±5.33^a^^,^^b^	212.32±32.45^a^^,^^b^

a P<0.05 *vs.* control group

b P<0.05 *vs.* HBV infection group

### Immune cells

The proportions and numbers of CD3^+^, CD4^+^, CD8^+^, CD4^+^/CD8^+^ and NK cells significantly declined in HIV/HBV coinfection group compared with those in control group (P<0.05), while they had no significant differences between control group and HBV infection group (P>0.05) (Table 3).

**Table 3:** Proportions and numbers of immune cells (x̅±s)

Index	Unit	Control group (n=86)	HBV infection group (n=86)	HIV/HBV coinfection group (n=86)
CD3+	%	72.43±5.94	71.32±5.45	65.12±4.23^a^
	Cells/µl	1645.39±180.4	1605.48±178.3	1258.32±132.34^a^
CD4+	%	43.42±7.52	42.43±6.53	35.74±3.43^a^
	Cells/µl	932.33±124.63	934.24±133.45	723.33±102.32^a^
CD8+	%	23.53±5.18	22.45±4.44	18.75±2.53^a^
	Cells/µl	548.65±187.65	536.23±187.4	448.56±94.54^a^
CD4+/CD8+	%	1.63±0.43	1.58±0.42	1.41±0.32^a^
NK	%	17.69±3.24	18.44±3.37	14.34±1.62^a^
	Cells/µl	439.49±83.49	449.24±81.67	384.32±58.49^a^

a P<0.05 *vs.* control group

### Inflammatory indices

The levels of IL-2, IL-6 and TNF-α were significantly higher, while the level of IL-10 was significantly lower in HBV infection group and HIV/HBV coinfection group than those in control group, and the above changes were more obvious in HIV/HBV coinfection group (P<0.05) (Table 4).

**Table 4:** Inflammatory indices (x̅±s)

Group	IL-2 (pg/mL)	IL-6 (pg/mL)	IL-10 (ng/mL)	TNF-α (ng/mL)
Control (n=86)	6.82±0.54	12.39±0.46	1.83±0.36	1.29±0.45
HBV infection (n=86)	10.29±1.18^a^	16.32±1.25^a^	1.23±0.24^a^	3.48±0.89^a^
HIV/HBV coinfection (n=86)	15.32±2.14^a^^,^^b^	19.38±2.04^a^^,^^b^	0.64±0.12^a^^,^^b^	8.42±2.12^a^^,^^b^

a P<0.05 *vs.* control group

b P<0.05 *vs.* HBV infection group

### Numbers of intestinal flora

The numbers of *Bifidobacterium, Lactobacillus* and *Bacteroides* significantly declined, while the numbers of *Enterobacter* and *Enterococcus* significantly rose in HBV infection group and HIV/HBV coinfection group compared with those in control group, and the above changes were more obvious in HIV/HBV coinfection group (P<0.05) (Table 5).

**Table 5:** Numbers of intestinal flora (log N/g, x̅±s)

Group	*Bifidobacterium*	*Lactobacillus*	*Bacteroides*	*Enterobacter*	*Enterococcus*
Control (n=86)	7.43±0.32	6.12±0.42	13.34±1.33	6.64±0.22	7.44±0.64
HBV infection (n=86)	6.32±0.45^a^	5.13±0.45^a^	9.57±1.23^a^	7.27±0.91^a^	8.34±0.86^a^
HIV/HBV coinfection (n=86)	5.12±0.32^a^^,^^b^	4.34±0.74^a^^,^^b^	8.85±0.32^a^^,^^b^	8.76±1.32^a^^,^^b^	9.53±1.42^a^^,^^b^

a P<0.05 *vs.* control group

b P<0.05 *vs.* HBV infection group

### Correlation analysis results of intestinal flora with liver function and immune function

The results of Pearson's correlation analysis revealed that the levels of IL-2, IL-6, IL-17, ALT, AST, GGT, DBiL and TDBi had significant negative correlations with *Bifidobacterium, Lactobacillus* and *Bacteroides*, but had significant positive correlations with *Enterobacter* and *Enterococcus* (P<0.05). The IL-10 level and the proportions of CD3^+^, CD4^+^, CD8^+^, CD4^+^/CD8^+^ and NK cells were significantly positively correlated with *Bifidobacterium, Lactobacillus* and *Bacteroides*, but significantly negatively correlated with *Enterobacter* and *Enterococcus* (P<0.05) (Table 6).

**Table 6:** Correlation analysis results of intestinal flora with liver function and immune function

Factor	*Bifidobacterium*		*Lactobacillus*		*Bacteroides*		*Enterobacter*		*Enterococcus*	

	r	*P*	r	*P*	r	*P*	r	*P*	r	*P*
IL-2	-1.787	0.014	-2.161	0.011	-1.548	0.019	1.732	0.015	0.636	0.034
IL-6	-0.943	0.029	-2.291	0.009	-2.622	0.005	1.849	0.014	0.973	0.028
IL-10	1.829	0.014	2.656	0.004	0.866	0.030	-2.888	0.002	-2.590	0.005
IL-17	-1.494	0.021	-0.036	0.043	-1.103	0.025	2.282	0.009	2.023	0.013
ALT	-2.348	0.009	-1.626	0.017	-1.475	0.021	2.363	0.008	0.127	0.040
AST	-0.702	0.033	-1.667	0.016	-2.364	0.007	1.461	0.022	1.018	0.027
GGT	-1.675	0.016	-2.552	0.006	-0.842	0.030	0.364	0.036	1.384	0.023
DBiL	-2.103	0.012	-1.526	0.019	-1.962	0.013	0.340	0.038	2.933	0.002
TDBi	-0.107	0.041	-1.552	0.018	-2.836	0.003	0.560	0.035	0.127	0.040
CD3+	0.728	0.031	1.964	0.013	2.807	0.003	-2.812	0.003	-2.722	0.004
CD4+	2.556	0.006	3.019	0.001	2.853	0.002	-1.758	0.015	-0.300	0.037
CD8+	1.033	0.027	0.518	0.036	0.304	0.037	-2.239	0.010	-1.504	0.020
CD4+/CD8+	2.949	0.001	0.755	0.031	1.375	0.023	-2.595	0.005	-1.557	0.018
NK	0.072	0.041	0.875	0.029	2.351	0.008	-1.137	0.025	-2.045	0.012

## Discussion

Due to the same route of transmission of HIV and HBV, the HIV/HBV coinfection rate is relatively high. HIV infection can alter the natural course of HBV infection, whose mechanism is that HIV destroys CD4^+^ T lymphocytes in patients through various ways, so that the HBV scavenging ability is weakened and the immune tolerance to HBV is produced in the body, thus making it unable to eliminate HBV. Therefore, patients with HIV/HBV coinfection are more prone to chronic and persistent high-level viremia in HBV, promoting the progression of hepatic fibrosis and raising the mortality rate of liver diseases^12^,^13^. In the present study, the results manifested that the levels of ALT, AST, GGT, DBiL and TDBi in HIV/HBV coinfection group were all higher than those in HBV infection group, indicating that HIV/HBV coinfection has a more significant impact on human liver function.

The changes in fecal microecology of HBV-infected patients are mainly manifested as the increase in pathogenic bacteria and decrease in beneficial bacteria^14^. Liver transplantation not only promotes the recovery of intestinal microecology, but also expands the diversity of intestinal microecology in patients with liver cirrhosis, similar to the intestinal microecology samples of normal people. HIV directly attacks lymphocytes or induces lymphocyte infiltration, and releases a variety of cytokines due to local immune activation, leading to intestinal epithelial cell damage, digestive dysfunction, increased gastrointestinal permeability, lipid metabolism disorders and decline in cell cycle regulation-related genes, and resulting in HIV-related bowel diseases^15^.

In the present study, the results revealed that the numbers of Bifidobacterium, Lactobacillus and Bacteroides were smaller, while the numbers of Enterobacter and Enterococcus were larger in HBV infection group than those in control group, and the above changes were more obvious in HIV/HBV coinfection group. It can be seen that the biological characteristics (type and number) of intestinal flora in patients with HIV/HBV coinfection are altered, in which the dominant intestinal bacteria represented by Lactobacillus, Bifidobacterium and Bacteroides are greatly decreased, and the pathogenic bacteria represented by Enterobacter and Enterococcus are greatly increased.

There are close relations between anatomy and function of the intestine and the liver. Healthy people have relatively stable structure of the intestinal flora. In the case of liver injury, gastrointestinal motility disorders and changes in intestinal permeability are usually accompanied, so that the pathogenic bacteria stay in the intestine for a longer time to reduce intestinal peristalsis and prolong the small intestine emptying time. As a result, pathogenic bacteria are more prone to adhesion and growth, and overgrown bacteria can secrete a large amount of cytotoxin, which suppresses the growth of beneficial bacteria, leads to intestinal epithelial lesions and promotes the colonization of intestinal pathogenic bacteria, thus resulting in intestinal flora disorders^16^.

In this study, the results of correlation analysis revealed that the levels of ALT, AST, GGT, DBiL and TDBi had significant negative correlations with Bifidobacterium, Lactobacillus and Bacteroides, but had significant positive correlations with Enterobacter and Enterococcus, also confirming the influence of the changes in intestinal flora on liver function.

The intestinal mucosal immune response in the body will be altered by the intestinal flora changes, mainly manifested as the antibody elimination and tolerance to antigens. Normal intestinal flora in healthy individuals will not induce immune responses^17^. After changes in intestinal flora, the probiotics will be reduced, destroying the local microecological balance. There is mutual influence and restraint between intestinal flora disorders and immune dysfunction, and once the immune tolerance is broken, it will persist throughout the pathological process of disease, ultimately leading to decline in immune function^18^.

As a bridge between the host flora and inflammatory diseases, Th17 can secrete pro-inflammatory factors such as IL-2 and IL-6, and also enhance the secretion of TNF-α, worsening the inflammatory response. Moreover, flora can induce the transformation of Th17 into Th1 and Th2 that play regulatory roles in inflammatory diseases. Bifidobacterium, through proteins and polypeptides such as fimbriae, peptidoglycan hydrolase and exopolysaccharides, interacts with human immune cells to regulate specific signalling pathways related to innate and adaptive immunity, promote the Th1-type immune response, lead to Th17 polarization and facilitate the differentiation of regulatory T cells (Tregs), thereby keeping the intestinal immunity^19^. Clostridium can induce the proliferation of Tregs and secrete IL-10, suppressing the inflammatory response in colitis mice^20^.

In the present study, the levels of IL-2, IL-6 and TNF-α were markedly higher, while the level of IL-10 was markedly lower in HIV/HBV coinfection group than those in HBV infection group and control group, suggesting that HIV worsens HBV-induced inflammatory response. The results of correlation analysis revealed that the levels of IL-2, IL-6 and IL-17 had significant negative correlations within fidobacterium, Lactobacillus and Bacteroides, but had significant positive correlations with Enterobacter and Enterococcus, while the level of IL-10 had the opposite correlations with the above bacteria. It can be inferred that intestinal flora disorders are involved in the inflammatory response.

T lymphocytes are involved in cellular immunity in the body and play auxiliary roles in some humoral immunity, which serve as the main functional cells in the immune surveillance system. The immunomodulatory effect of T cells is mainly realized by CD4^+^ and CD8^+^. CD4^+^ coordinates the differentiation of B cells to produce antibodies, which is an important player in the identification and elimination of pathogen-infected cells. Meanwhile, CD4^+^ is an important receptor of HIV and also the main target of attack of HIV. Therefore, the number of CD4^+^ T lymphocytes is an important basis for predicting the progression of disease in HIV-infected patients^21^. Besides, CD8+ restrains the synthesis and secretion of antibodies and the proliferation of T cells, and acts as a key player in the anti-tumor immune response^22^.

The levels of CD4^+^ and CD8^+^ and the CD4^+^/CD8^+^ ratio are key indices for measuring the cellular immune status. In this study, no statistically significant differences were found in NK cells, CD4+, CD8+ and CD4+/CD8+ between control group and HBV infection group, inconsistent with previous studies. The levels of NK cells, CD4^+^, CD8^+^ and CD4^+^/CD8^+^ in HIV/HBV coinfection group were far higher than those in the other two groups. As for the reason why, there was no difference in the immune function between the two groups, only T lymphocytes and NK cells were detected, and neither B lymphocytes nor humoral immunoglobulins were detected. Moreover, the level of local immune response caused by intestinal structure and function changes may be lower than the system detectable level. In addition, it was found that cellular immunity was negatively correlated with Enterobacter and Enterococcus, but positively correlated with Bifidobacterium, Lactobacillus and Bacteroides. The above findings demonstrate that immunoregulation of intestinal flora is not sufficient to affect the systemic cellular immunity, and also indirectly indicate that the influencing factors for the immune function of patients with HIV/HBV coinfection may be affected by the collective effects of intestinal microecology changes and other factors.

The present results provide further insights into the dysbiosis of the intestinal microbiota in patients with human immunodeficiency virus and hepatitis B virus-induced chronic liver disease and might potentially serve as guidance for the probiotics interventions of these diseases. The gut microbiome may represent fertile targets for prevention or management of HBV-induced chronic liver disease. Fecal microbiota transplantation may be a useful therapy for HBV-related disease in the future. The concept of the “gut-liver axis” and the progression in intestinal microecology is considered crucial.

In conclusion, there are abnormalities in the intestinal flora, liver function, inflammatory factors and immune cells in patients with HIV/HBV coinfection. HIV worsens the liver damage and immuno-inflammatory response in HBV patients, and the intestinal flora indirectly affects the liver function and immuno-inflammatory response. Nevertheless, this study still has some limitations. For example, the sample size was small. Moreover, the changes in intestinal flora, liver function and immuno-inflammatory indices during clinical treatment in patients with HIV/ HBV coinfection were not explored. Hence, in-depth studies with larger sample sizes are in need in the future.
